# The Protective Effect of CBD in a Model of In Vitro Ischemia May Be Mediated by Agonism on TRPV2 Channel and Microglia Activation

**DOI:** 10.3390/ijms232012144

**Published:** 2022-10-12

**Authors:** Daniele Lana, Elisa Landucci, Costanza Mazzantini, Giada Magni, Domenico Edoardo Pellegrini-Giampietro, Maria Grazia Giovannini

**Affiliations:** 1 Department of Health Sciences, Section of Clinical Pharmacology and Oncology, University of Florence, Viale Pieraccini 6, 50139 Firenze, Italy; 2 Institute of Applied Physics “Nello Carrara”, National Research Council (IFAC-CNR), Via Madonna del Piano 10, 50019 Sesto Fiorentino, Italy

**Keywords:** cannabidiol, Δ-9-tetrahydrocannabinol, ischemia, neuroprotection, microglia, astrocytes, organotypic hippocampal slices, TRPV2, confocal microscopy, rod microglia

## Abstract

Cannabinoids, used for centuries for recreational and medical purposes, have potential therapeutic value in stroke treatment. Cannabidiol (CBD), a non-psychoactive compound and partial agonist of TRPV2 channels, is efficacious in many neurological disorders. We investigated the effects of CBD or Δ9-tetrahydrocannabinol (THC) in rat organotypic hippocampal slices exposed to oxygen-glucose deprivation (OGD), an in vitro model of ischemia. Neuronal TRPV2 expression decreased after OGD, but it increased in activated, phagocytic microglia. CBD increased TRPV2 expression, decreased microglia phagocytosis, and increased rod microglia after OGD. THC had effects contrary to those of CBD. Our results show that cannabinoids have different effects in ischemia. CBD showed neuroprotective effects, mediated, at least in part, by TRPV2 channels, since the TRPV2 antagonist tranilast blocked them, while THC worsened the neurodegeneration caused by ischemia. In conclusion, our results suggest that different cannabinoid molecules play different roles in the mechanisms of post-ischemic neuronal death. These different effects of cannabinoid observed in our experiments caution against the indiscriminate use of cannabis or cannabinoid preparations for recreational or therapeutic use. It was observed that the positive effects of CBD may be counteracted by the negative effects caused by high levels of THC.

## 1. Introduction

Cerebral ischemia occurs as a result of a reduction in cerebral blood flow and blockade of oxygen and nutrient supply to the downstream cerebral tissue, leading to neurodegeneration and neuronal death. Cerebral ischemia is one of the major causes of death or disability in the aged Western population [1]. The only therapeutical option so far available to treat cerebral ischemia is the thrombolytic drug tPA. A neuroprotective treatment, efficacious if administered after the onset of the ischemic insult, still remains to be developed. Understanding the pathophysiological bases for neurodegeneration after ischemia is a necessary area of background study before we will be able to identify new targets and plan new therapeutic interventions. 

For centuries, cannabis (*Cannabis sativa*) has been used not only as a recreational drug but also as a natural medicine. The role of cannabis and its active compounds (cannabinoids) in medicine is rapidly evolving since accumulating preclinical studies suggest that cannabinoids have potential therapeutic value in stroke. In Italy, at present, cannabis can be prescribed for specific therapeutic purposes, as reported in the Ministerial Decree of 9/11/2015 [2]. Nevertheless, cannabis and several cannabinoid-based medications have concerning side-effect profiles, which may limit their use in certain patient populations [3]. The two most rigorously studied phytocannabinoids are Δ9-tetrahydrocannabinol (THC) and cannabidiol (CBD). THC accounts for memory impairment, sedation, hyperphagia, and abuse potential typical of cannabis use, whereas CBD lacks all of these typical “THC-like” properties [4], particularly the abuse potential. CBD reduces brain edema and blood–brain barrier permeability associated with ischemia [5], increases cerebral blood flow [6], and reduces infarct size when administered ≤ 6 h after stroke [7]. Repeated treatment with CBD improves functional outcome and survival rates after stroke, suggesting that CBD may have neuroprotective effects at the early phase and late time points. On the contrary, it has been shown that recreational use of cannabis is associated with a 2.25-fold increase in the risk of acute ischemic stroke among people aged 25 to 34 years [8], posing a threat to health through the illegal use of cannabis among the young population. In a recent review [9], Chesney and colleagues (2020) tried to determine whether the published literature demonstrates that CBD may have significant pharmacological and symptomatic effects at the doses found in OTC preparations. It was found that most of the evidence of CBD’s beneficial effects derives from studies of pure, pharmaceutical-grade CBD at relatively high doses. Relatively few studies examined the effect of OTC preparations in which compounds other than CBD are present [9]. Accordingly, there is so far little evidence that OTC products containing CBD have health benefits, and their safety has not been investigated. Controlled trials of OTC and low-dose CBD preparations are needed to resolve these issues.

While it is known that THC is a CB1R and CB2R partial agonist [10,11], the pharmacology of CBD is still to be completely defined. CBD has low affinity for CB1 and CB2 receptors [11], and can operate through other receptors and pathways. Indeed, CBD is also a partial agonist of TRPV1 and TRPV2 channels [12,13], and it increases intracellular Ca^2+^ in dorsal root ganglion neurons via the activation of TRPV2 channels [14]. A docking experiment analyzed the interaction of CBD with TRPV1 and TRPV2 and showed that CBD may preferentially interfere with the latter [15]. It has been demonstrated that CBD is the most potent and efficacious phytocannabinoid acting on TRPV2 receptors [12,14]. It has also been demonstrated that in the hippocampus, TRPV2 channels are expressed on neurons [16,17,18], contributing to the maintenance of neuronal homeostasis [19,20,21]. CBD has further been shown to have central anti-inflammatory properties [22], and to have protective effects on CA1 neurodegeneration caused by OGD, mediated, at least in part, by the TRPV2 channel [23]. The purpose of the present study was to investigate the effects of CBD compared to THC on neuronal degeneration, focusing on TRPV2 involvement, glia activation, and microglia phagocytosis. The TRPV2 channel’s subfamily, members of the transient receptor potential (TRP) channels, are expressed in the brain in neurons, astrocytes, and microglia, where they mediate many different functions. It is of great interest that the activation of TRPV2 by CBD, but not by other cannabinoids, increases phagocytic activity in microglia in culture [24]. 

## 2. Results

### 2.1. Effects of CBD on Neural Degeneration and TRPV2 Expression in CA1 Hippocampus after OGD

**Figure 1:**
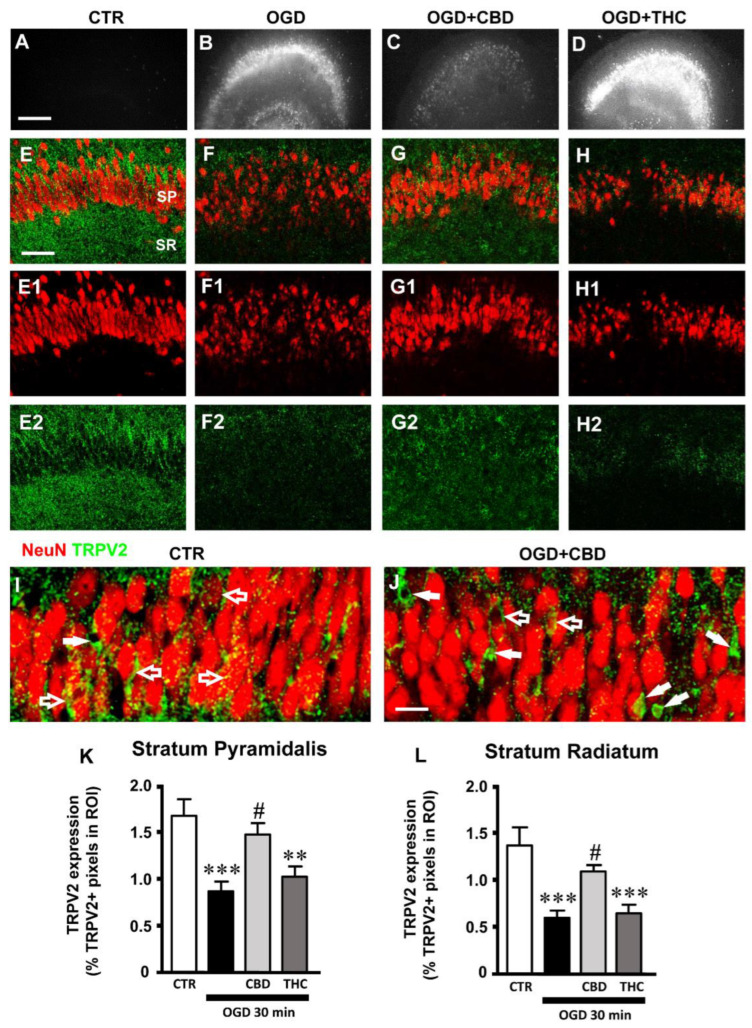
(**A**–**D**) Representative images of PI fluorescence in control (**A**), OGD (**B**), OGD + CBD, and OGD+ THC slices. Scale bar: 200 µm. (**E**–**H2**) Representative confocal images of immunostaining of neurons (NeuN, red) and TRPV2 channels (TRPV2, green) in CA1 SP and SR of CTR (**E**-**E2**), OGD (**F**–**F2**), OGD + CBD (**G**–**G2**), and OGD + THC (**H**–**H2**) slices. Images are z-projections of a single confocal z-scan captured with a 20× objective (total thickness: 1.2 µm). Scale bar: 75 µm. (**I**–**J**) Representative confocal images of immunostaining of neurons (NeuN, red) and TRPV2 channels (green) in CA1 SP of a CTR (**I**) and an OGD + CBD slice (**J**). Images are z-projections of a single confocal z-scan captured with a 40× objective (total thickness: 0.6 µm). Scale bar: 20 µm. (**I**) In CTR slices, the expression of TRPV2 was localized mainly on pyramidal neurons (open arrows). (**J**) In OGD + CBD slices, the expression of TRPV2 was localized mainly on non-neuronal cells (white arrows). (**K**) Quantitative analyses of TRPV2 channel expression in CA1 SP of CTR (n = 11), OGD (n = 10), OGD + CBD (n = 9), and OGD + THC slices (n = 9). Statistical analysis: one-way ANOVA *p* < 0.001; Newman–Keuls post hoc test *** *p* < 0.001 and ** *p* < 0.01 vs. CTR, # *p* < 0.05 vs. OGD. (**L**) Quantitative analyses of TRPV2 channel expression in CA1 SR of CTR (n = 12), OGD (n = 10), OGD + CBD (n = 8), and OGD + THC slices (n = 10). Statistical analysis: one-way ANOVA *p* < 0.001; Newman–Keuls post hoc test *** *p* < 0.001 vs. CTR, # *p* < 0.05 vs. OGD. Data reported in the graphs are expressed as the mean ± SEM.

We had previously demonstrated that an OGD insult causes degeneration and morphological alterations of CA1 pyramidal neurons, as well as tissue disorganization [23]. Here, we further assessed the effects of CBD or THC administration on the viability of CA1 pyramidal neurons and on TRPV2 channel expression on organotypic hippocampal slices harvested 24 h after 30 min OGD. Organotypic slices of the four experimental groups (CTR, OGD, OGD + CBD, OGD + THC) were immunostained for the TRPV2 channel and for NeuN to visualize the neurons (Figure 1E–H2). As previously published [23], we confirmed here, qualitatively by PI fluorescence (Figure 1A–D), that 24 h after OGD, CA1 pyramidal neurons showed signs of degeneration (Figure 1B). Indeed, OGD increased neuronal death in CA1 SP, caused modifications of neuronal morphology such as pyknosis and karyorrhexis, and brought about disorganization of the hippocampal tissue (Figure 1B,F–F1). CBD significantly reverted all these degenerative effects caused by OGD (Figure 1C,G–G1), while THC had no significant effect (Figure 1D,H–H1). Furthermore, the TRPV2 antagonist TNL (50 µM, OGD + CBD + TNL) significantly reverted the neuroprotective effect of CBD after OGD. Indeed, after OGD, we found a highly significant increase (+1500% vs. controls) in karyorrhectic neurons (low-density nucleus (LDN) neurons), and CBD significantly prevented their formation (+ 62% vs. controls). However, in OGD + CBD + TNL-treated slices, the number of damaged neurons was significantly higher than in OGD slices (+ 170% vs. OGD slices). Statistical analysis demonstrated that the effect of tranilast on LDN neurons’ density was significant not only vs. OGD + CBD slices but also in comparison with OGD slices (one-way ANOVA: F3,34 = 18.11; *p*-value < 0.0001; Newman–Keuls multiple comparisons test: *** *p* < 0.001 OGD vs. CTR; *** *p* < 0.001 OGD + CBD + TNL vs. OGD + CBD; # *p* < 0.05 OGD + CBD + TNL vs. OGD). All these data taken together confirmed that in organotypic hippocampal slices, CBD had a significant neuroprotective effect on CA1 pyramidal neurons damaged by OGD, confirming the data published by Landucci and colleagues (2021) [23]. Furthermore, the protective effect of CBD was significantly prevented by the TRPV2 antagonist TNL, indicating that TRPV2 channels play a role in shaping the effect of CBD. 

Qualitative analysis of the immunostaining of the TRPV2 channel showed that in control slices, TRPV2 channels were expressed in both the SP and SR of the CA1 hippocampus, as shown in Figure 1E–E2. As evidenced in the confocal image taken at a higher magnification (Figure 1I), in the CA1 SP of control slices, the TRPV2 immunostaining was mainly localized to pyramidal neurons (Figure 1I, open arrows). The qualitative and quantitative analyses of the expression levels of the TRPV2 channel in CA1 SP showed that OGD caused a significant decrease in its expression, in comparison to control slices (Figure 1E–E2,F–F2,K). The treatment with CBD significantly prevented the OGD-induced decrease in TRPV2 channel expression, which was not significantly different from control slices (Figure 1G-G2,K). On the contrary, in the SP of THC-treated slices, the expression of the TRPV2 channel was not significantly different from that of OGD slices (Figure 1H–H2,K; statistical analysis: one-way ANOVA: F3,35 = 7.912; *p*-value = 0.0004; Newman–Keuls multiple comparisons test: *** *p* < 0.001 vs. CTR; ** *p* < 0.01 vs. CTR; # *p* < 0.05 vs. OGD).

The qualitative and quantitative analyses of the expression levels of the TRPV2 channel in the SR of the CA1 hippocampus showed a significant decrease in TRPV2 expression in the slices exposed to OGD, compared to control slices (Figure 1E–E2,F–F2,L). In the SR of OGD slices treated with CBD, we found that CBD prevented the OGD-induced decrease in TRPV2 channel expression in comparison to OGD slices (Figure 1G–G2,L). On the contrary, in the SR of THC-treated slices, the expression of the TRPV2 channel was not significantly different from that of OGD slices (Figure 1H–H2,L; statistical analysis: one-way ANOVA: F3,36 = 8.258; *p*-value = 0.0003; Newman–Keuls multiple comparisons test: *** *p* < 0.001 vs. CTR; # *p* < 0.05 vs. OGD).

When comparing the immunostaining of the TRPV2 channel in the SP of the four experimental groups, we observed that in CTR slices, the expression of TRPV2 was localized mainly to pyramidal neurons, as shown in the representative confocal images of Figure 1I (open arrows). In OGD slices treated with CBD, the expression of TRPV2 was localized mainly to non-neuronal cells (Figure 1J, white arrows).

To verify which cell type other than neurons might express the TRPV2 channel in CA1 SP after OGD, we performed further qualitative and quantitative analyses to colocalize TRPV2 immunostaining with astrocytes and microglia. 

### 2.2. Alterations of Astrocytes in Stratum Pyramidale (SP) and Stratum Radiatum (SR) of the CA1 Hippocampal Region after OGD

**Figure 2:**
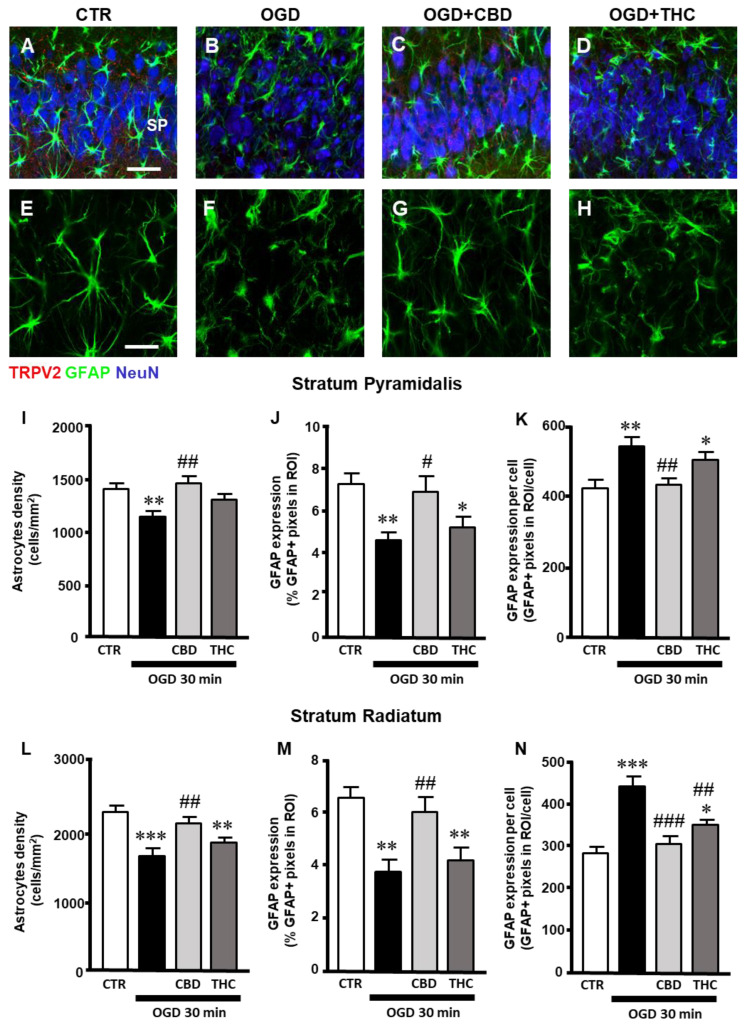
(**A**–**D**) Representative confocal images of immunostaining of TRPV2 channels (TRPV2, red), astrocytes (GFAP, green), and neurons (NeuN, blue) in CA1 SP and SR of CTR (**A**), OGD (**B**), OGD + CBD (**C**), and OGD + THC (**D**) slices. Images are z-projections of 10 confocal z-scans captured with a 40× objective (total thickness: 6 µm). Scale bar: 40 µm. (**A**–**D**) No colocalization of TRPV2 channel with astrocytes is present in the four experimental groups. (**E**–**H**) Enlargements of confocal images in panels (**A**–**D**) of astrocytes’ immunostaining (GFAP, green). In OGD slices (**F**), astrocytes changed their morphology, becoming clasmatodendrotic, with shorter, thicker, and twisted branches compared to astrocytes from control slices (**E**). Treatment with CBD prevented the clasmatodendrotic modifications of astrocytes (**G**), while THC had no significant effect (**H**). (**I**) Quantitative analyses of astrocytes’ density in CA1 SP of CTR (n = 11), OGD (n = 11), OGD + CBD (n = 12), and OGD + THC slices (n = 10). Statistical analysis: one-way ANOVA *p* < 0.01; Newman–Keuls post hoc test ** *p* < 0.01 vs. CTR, ## *p* < 0.01 vs. OGD. (**J**) Quantitative analyses of GFAP expression in CA1 SP of CTR (n = 11), OGD (n = 10), OGD + CBD (n = 10), and OGD + THC slices (n = 10). Statistical analysis: one-way ANOVA *p* < 0.01; Newman–Keuls post hoc test ** *p* < 0.01 and * *p* < 0.05 vs. CTR, # *p* < 0.05 vs. OGD. (**K**) Quantitative analyses of GFAP expression per cell in CA1 SP of CTR (n = 10), OGD (n = 11), OGD + CBD (n = 11), and OGD + THC slices (n = 9). Statistical analysis: one-way ANOVA *p* < 0.01; Newman–Keuls post hoc test ** *p* < 0.01 and * *p* < 0.05 vs. CTR, ## *p* < 0.01 vs. OGD. (**L**) Quantitative analyses of astrocytes’ density in CA1 SR of CTR (n = 10), OGD (n = 12), OGD + CBD (n = 9), and OGD + THC slices (n = 9). Statistical analysis: one-way ANOVA *p* = 0.0001; Newman–Keuls post hoc test *** *p* < 0.001 and ** *p* < 0.01 vs. CTR, ## *p* < 0.01 vs. OGD. (**M**) Quantitative analyses of GFAP expression in CA1 SR of CTR (n = 11), OGD (n = 9), OGD + CBD (n = 10), and OGD + THC slices (n = 10). Statistical analysis: one-way ANOVA *p* < 0.001; Newman–Keuls post hoc test ** *p* < 0.01 vs. CTR, ## *p* < 0.01 vs. OGD. (**N**) Quantitative analyses of GFAP expression per cell in CA1 SR of CTR (n = 11), OGD (n = 9), OGD + CBD (n = 9), and OGD + THC slices (n = 9). Statistical analysis: one-way ANOVA *p* < 0.0001; Newman–Keuls post hoc test *** *p* < 0.001 and * *p* < 0.05 vs. CTR, ### *p* < 0.001 and ## *p* < 0.01 vs. OGD.

Triple-labeling fluorescence immunohistochemistry of the TRPV2 channel (red), NeuN to visualize neurons (blue), and GFAP to visualize astrocytes on organotypic slices of the four experimental groups (CTR, OGD, OGD + CBD, OGD + THC) is shown in Figure 2A–D. A qualitative analysis indicated that no colocalization of the TRPV2 channel with astrocytes occurred in the four experimental groups, Figure 2E–H). 

Nevertheless, the qualitative analysis of GFAP-positive astrocytes in CA1 SP and SR of the four experimental groups (Figure 2E–H) showed that, 24 h after OGD, astrocytes changed their morphology, becoming clasmatodendrotic, with shorter, thicker, and twisted branches in comparison to astrocytes from control slices. This morphofunctional modification has been shown to depend on the acidification of the parenkyma after ischemia [25,26]. From the qualitative images, it appears that treatment with CBD prevented the clasmatodendrotic modifications of astrocytes (Figure 2G), while THC had no significant effect (Figure 2H). Quantitative analyses of the density of astrocytes (Figure 2I,L), the expression of GFAP (Figure 2J,M), and the expression of GFAP/astrocyte (Figure 2K,N) were performed separately in the SP and SR of the CA1 hippocampus for the four experimental groups. In the CA1 SP of organotypic hippocampal slices exposed to OGD, the density of astrocytes was lower than in control slices. Treatment with CBD significantly prevented the OGD-induced decrease in astrocytes, while THC had no effect (Figure 2I; statistical analysis: one-way ANOVA: F3,40 = 6.058; *p*-value = 0.0017; Newman–Keuls multiple comparisons test: ** *p* < 0.01 vs. CTR; ## *p* < 0.01 vs. OGD). In OGD slices, GFAP protein expression was significantly decreased from the basal levels found in control slices, and CBD prevented this effect while THC did not (Figure 2J; statistical analysis: one-way ANOVA: F3,37 = 5.510; *p*-value = 0.0031; Newman–Keuls multiple comparisons test: ** *p* < 0.01 vs. CTR; * *p* < 0.05 vs. CTR; # *p* < 0.05 vs. OGD). Interestingly, the levels of GFAP protein/astrocyte increased significantly in OGD slices, indicating that astrocytes, although less numerous, were in an activated form 24 h after ischemia. CBD prevented this effect, while THC did not (Figure 2K; statistical analysis: one-way ANOVA: F3,37 = 6.495; *p*-value = 0.0012; Newman–Keuls multiple comparisons test: ** *p* < 0.01 vs. CTR; * *p* < 0.05 vs. CTR; ## *p* < 0.01 vs. OGD).

Similar results were obtained in CA1 SR. The density of astrocytes decreased in the SP of organotypic hippocampal slices exposed to OGD in comparison to control slices. In OGD + CBD slices, CBD prevented the OGD-induced decrease in astrocytes’ density, while THC had no effect (Figure 2L; statistical analysis: one-way ANOVA: F3,36 = 9.229; *p*-value = 0.0001; Newman–Keuls multiple comparisons test: *** *p* < 0.001 vs. CTR; ** *p* < 0.01 vs. CTR; ## *p* < 0.01 vs. OGD). In OGD slices, GFAP protein expression decreased from the basal levels found in control slices, and CBD prevented this effect while THC did not (Figure 2M; statistical analysis: one-way ANOVA: F3,36 = 8.239; *p*-value = 0.0003; Newman–Keuls multiple comparisons test: ** *p* < 0.01 vs. CTR; ## *p* < 0.01 vs. OGD). The levels of GFAP protein/astrocytes increased significantly in OGD exposed slices, indicating that astrocytes, although fewer in number, were activated 24 h after ischemia. CBD prevented this effect, while THC did not (Figure 2N; statistical analysis: one-way ANOVA: F3,34 = 16.24; *p*-value = 0.0001; Newman–Keuls multiple comparisons test: *** *p* < 0.001 vs. CTR; * *p* < 0.05 vs. CTR; ### *p* < 0.001 vs. OGD; ## *p* < 0.01 vs. OGD).

### 2.3. Expression of TRPV2 Channel in Phagocytic Microglia in Stratum Pyramidale (SP) of CA1 Hippocampal Region

**Figure 3:**
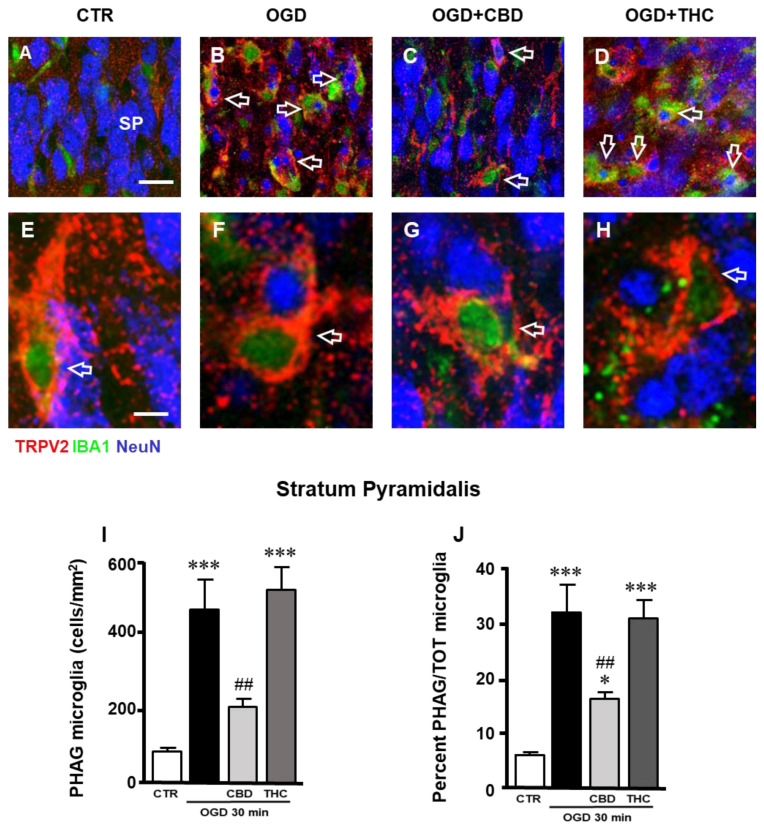
(**A**–**D**) Representative confocal images of immunostaining of TRPV2 channels (TRPV2, red), total microglia (IBA1, green), and neurons (NeuN, blue) in CA1 SP of CTR (**A**), OGD (**B**), OGD + CBD (**C**), and OGD + THC (**D**) slices. Images are z-projections of two confocal z-scans captured with a 63× objective (total thickness: 0.6 µm). Scale bar: 20 µm. Twenty-four hours after OGD, the expression of TRPV2 channel in CA1 SP was mainly localized on activated microglia cells that showed a green cellular body and yellow-orange cytoplasm and projections (open arrows in panel **B**) that phagocytose neurons or neuronal fragments (blue cells and cellular fragments within the microglia cell body in panel **B**). From a qualitative analysis, it appears that this effect was prevented by CBD (open arrows in panel **C**), but not by THC (open arrows in panel **D**). (**E**–**H**) Images are z-projections of five confocal z-scans captured with a 63× objective (total thickness: 1.5 µm). Scale bar: 10 µm. These enlargements clearly show the expression of the TRPV2 channel in activated microglia (open arrows in panels **E**–**H**). (**I**) Quantitative analyses of phagocytic microglia density in CA1 SP of CTR (n = 8), OGD (n = 8), OGD + CBD (n = 8), and OGD + THC slices (n = 7). Statistical analysis: one-way ANOVA *p* < 0.0001; Newman–Keuls post hoc test *** *p* < 0.001 vs. CTR, ## *p* < 0.01 vs. OGD. (**J**) Quantitative analyses of percentage of phagocytic microglia out of the total microglia in CA1 SP of CTR (n = 8), OGD (n = 8), OGD + CBD (n = 8), and OGD + THC slices (n = 7). Statistical analysis: one-way ANOVA *p* < 0.0001; Newman–Keuls post hoc test *** *p* < 0.001 and * *p* < 0.05 vs. CTR, ## *p* < 0.05 vs. OGD.

Triple-labeling fluorescence immunohistochemistry of neurons (NeuN, blue), microglia (IBA1, green), and TRPV2 (red) (Figure 3A–D) showed that 24 h after OGD, the expression of TRPV2 in CA1 SP was mainly localized on activated microglia cells (Figure 3B, cells with a green cellular body and yellow-orange projections, open arrows) that phagocytose neurons or neuronal fragments (Figure 3B, blue cells and cellular fragments inside microglia). Through qualitative analysis, it appeared that this effect was prevented by CBD (Figure 3C), but not by THC (Figure 3D). Enlarged images, shown in Figure 3E–H, demonstrate colocalization of the TRPV2 channel with phagocytic microglia cells in CTR, OGD, OGD + CBD, and OGD + THC slices. Although phagocytic TRPV2-positive microglia cells are present in the SP of all the experimental groups (Figure 3E–H), the quantitative analysis demonstrated that OGD significantly increased the number of phagocytic microglia, and CBD significantly reverted this effect while THC did not (Figure 3I; statistical analysis: one-way ANOVA: F3,27 = 16.46; *p*-value = 0.0001; Newman–Keuls multiple comparisons test: *** *p* < 0.001 vs. CTR; ## *p* < 0.01 vs. OGD). By expressing phagocytic microglia as a percentage of the total microglia, we demonstrated that about 30% of microglia in the SP of OGB and of OGD + THC slices were in an activated, phagocytic form, while this percentage was only 15% in OGD + CBD slices, possibly because the neuronal damage was less intense in CBD-exposed slices after OGD (Figure 3J; statistical analysis: one-way ANOVA: F3,27 = 16.42; *p*-value = 0.0001; Newman–Keuls multiple comparisons test: *** *p* < 0.001 vs. CTR; * *p* < 0.05 vs. CTR; ## *p* < 0.01 vs. OGD).

### 2.4. Microglia Morphofunctional Modifications after OGD in Stratum Pyramidale (SP) and Stratum Radiatum (SR) of CA1 Hippocampus

**Figure 4:**
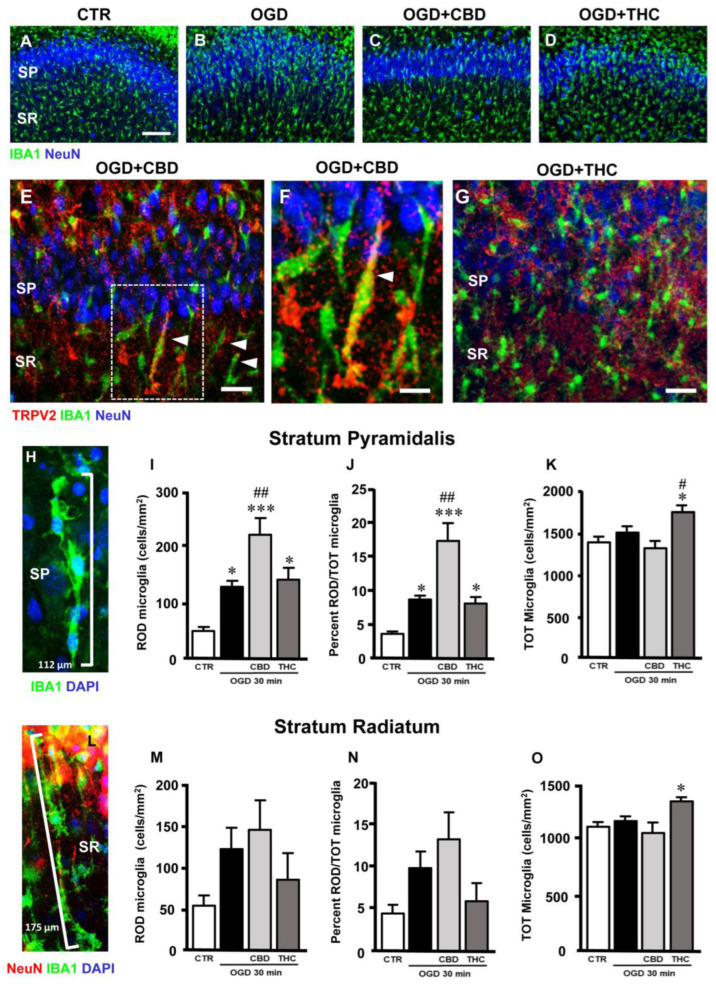
(**A**–**D**) Representative confocal images of immunostaining of neurons (NeuN, blue) and total microglia (IBA1, green) in CA1 SP and SR of CTR (**A**), OGD (**B**), OGD + CBD (**C**), and OGD + THC (**D**) slices. Images are z-projections of a single confocal z-scan captured with a 20× objective (total thickness: 1.2 µm). Scale bar: 75 µm. In OGD slices, many microglia acquired a rod-like morphology, with elongated cell bodies ranging from 40 µm to over 100 µm, and with their longer axis perpendicular to CA1 SP, while other microglia acquired a round morphology typical of activated microglia. (**E**–**G**) Representative confocal images of immunostaining of TRPV2 channels (TRPV2, red), total microglia (IBA1, green), and neurons (NeuN, blue) in CA1 SP and SR of an OGD + CBD (**E**,**F**) and an OGD + THC slice (**G**). Images are z-projections of three confocal z-scans captured with a 40× objective (total thickness: 1.8 µm). Scale bar for panels E,G: 20 µm. Scale bar for panel F: 10 µm. In OGD + CBD slices, TRPV2 was expressed in many rod microglia (arrowheads in panel E), as better evidenced in panel F (enlargement of the framed area in panel E). Rod microglia cells are much less evident in the OGD + THC slices (panel G). (**H**) Representative confocal images of immunostaining of total microglia (IBA1, green) and DAPI nuclear staining (DAPI, blue) in CA1 SP of an OGD + CBD slice. Images are z-projections of 10 confocal z-scans captured with a 63× objective (total thickness: 3 µm). Four rod microglia cells (evidenced by the presence of DAPI-positive nuclei in blue) form a 112 µm-long train that stretches throughout the SP. (**I**) Quantitative analyses of rod microglia density in CA1 SP of CTR (n = 6), OGD (n = 8), OGD + CBD (n = 8), and OGD + THC (n = 7) slices. Statistical analysis: one-way ANOVA *p* < 0.0001; Newman–Keuls post hoc test *** *p* < 0.001 and * *p* < 0.05 vs. CTR, ## *p* < 0.01 vs. OGD. (**J**) Quantitative analyses of rod microglia expressed as percentage of total microglia in CA1 SP of CTR (n = 6), OGD (n = 8), OGD + CBD (n = 7), and OGD + THC (n = 7) slices. Statistical analysis: one-way ANOVA *p* < 0.0001; Newman–Keuls post hoc test *** *p* < 0.001 and * *p* < 0.05 vs. CTR, ## *p* < 0.05 vs. OGD. (**K**) Quantitative analyses of total microglia density in CA1 SP of CTR (n = 6), OGD (n = 8), OGD + CBD (n = 8), and OGD + THC (n = 7) slices. Statistical analysis: one-way ANOVA *p* < 0.01; Newman–Keuls post hoc test * *p* < 0.05 vs. CTR, # *p* < 0.05 vs. OGD. (**L**) Representative confocal images of immunostaining of neurons (NeuN, red), total microglia (IBA1, green), and DAPI nuclear staining (DAPI, blue) in CA1 SP of an OGD + THC slice. Images are z-projections of 10 confocal z-scans captured with a 63× objective (total thickness: 3 µm). Five rod microglia cells form a 175 µm-long train that spans from the SR into the SP. The trains are often adjacent to apical dendrites of pyramidal neurons that project into the SR (shown in red). (**M**) Quantitative analyses of rod microglia density in CA1 SR of CTR (n = 6), OGD (n = 5), OGD + CBD (n = 8), and OGD + THC slices (n = 5). Statistical analysis: one-way ANOVA *p* = 0.1549 n.s. (**N**) Quantitative analyses of rod microglia expressed as percentage of total microglia in CA1 SR of CTR (n = 6), OGD (n = 5), OGD + CBD (n = 8), and OGD + THC (n = 5) slices. Statistical analysis: one-way ANOVA *p* = 0.0682 n.s. (**O**) Quantitative analyses of total microglia density in CA1 SR of CTR (n = 6), OGD (n = 5), OGD + CBD (n = 8), and OGD + THC slices (n = 5). Statistical analysis: one-way ANOVA *p* < 0.05; Newman–Keuls post hoc test * *p* < 0.05 vs. CTR.

Immunostaining of total microglia with anti-IBA1 antibody showed that OGD caused significant modifications of many microglia cells, which acquired a round morphology typical of activated microglia. Furthermore, many microglia acquired a rod-like morphology, with elongated cell bodies ranging from 40 µm to over 100 µm long, and with their longer axis perpendicular to CA1 SP (Figure 4A–H,L; see also [27]). 

Confirming previous findings in other brain regions and different types of insult [28,29], and from our own laboratory [27], we observed that rod microglia align end-to-end one after another to form trains (Figure 4H,L). As an example, we show in Figure 4H that four rod microglia cells (evidenced by the presence of DAPI-positive nuclei in blue) form a 112 µm-long train that stretches throughout the SP (SP neurons are not shown for clarity). In Figure 4L, we show five rod microglia cells that form a 175 µm-long train that spans from the SR into the SP. The trains are often adjacent to apical dendrites of pyramidal neurons that project into the SR (shown in red in Figure 4L).

We carried out a quantitative analysis of rod microglia in the control, OGD, OGD + CBD, and OGD + THC slices. Rod microglia were defined as IBA1-positive cells longer than 40 µm. As shown in Figure 4I, the density of rod microglia increased significantly in the CA1 SP of OGD slices (+155% vs. controls), OGD + CBD slices (+342% vs. controls), and OGD + THC slices (+182.7% vs. controls) (Figure 4I; statistical analysis: one-way ANOVA: F3,25 = 11.22; *p*-value = 0.0001; Newman–Keuls multiple comparisons test: * *p* < 0.05 vs. CTR; *** *p* < 0.001 vs. CTR; ## *p* < 0.01 vs. OGD). 

In addition, rod microglia, expressed as the percentage of total microglia, increased significantly in the CA1 SP of OGD (+155% vs. controls), OGD + CBD (+342% vs. controls), and OGD + THC slices (+182.7% vs. controls) (Figure 4J; statistical analysis: one-way ANOVA: F3,25 = 11.22; *p*-value = 0.0001; Newman–Keuls multiple comparisons test: * *p* < 0.05 vs. CTR; *** *p* < 0.001 vs. CTR; ## *p* < 0.01 vs. OGD). 

It is interesting to note that in OGD + CBD slices, TRPV2 was expressed in many rod microglia (Figure 4E, arrowheads), as better evidenced in Figure 4F (enlargement of the framed area in Figure 4E). Rod microglia cells were much less evident in the OGD + THC slices (Figure 4G). 

In the CA1 SR, rod microglia increased, although not significantly, in OGD, OGD + CBD, and OGD + THC slices (Figure 4M; statistical analysis: one-way ANOVA: F3,20 = 1.945; *p*-value = 0.155, n.s.). In the SR, rod microglia, expressed as the percentage of the total microglia, increased, although not significantly, in OGD, OGD + CBD, and OGD + THC slices (Figure 4N; statistical analysis: one-way ANOVA: F3,20 = 1.945; *p*-value = 0.155, n.s.).

As shown Figure 4K,O, the density of total IBA1-positive microglia increased significantly only in OGD + THC-treated slices in both the SP (Figure 4K; statistical analysis: one-way ANOVA: F3,25 = 5.43; *p*-value = 0.0051; Newman–Keuls multiple comparisons test: * *p* < 0.05 vs. CTR) and SR (Figure 4O; statistical analysis: one-way ANOVA: F3,20 = 2.821; *p*-value = 0.051; Newman–Keuls multiple comparisons test: * *p* < 0.05 vs. CTR; *** *p* < 0.001 vs. CTR; ## *p* < 0.01 vs. OGD). No significant differences of total microglia density were found in the SP or SR among the control, OGD, or OGD + CBD-treated slices (Figure 4K,O), thus demonstrating that the increase in rod microglia after OGD was not due to an increase in total microglia cells, but to morphological modifications of existing microglia, confirming previous findings that showed that rod microglia originate from morphofunctional modifications of resident CNS microglia [27].

## 3. Discussion

Medical cannabis and individual cannabinoids, such as cannabidiol (CBD) and D9-tetrahydrocannabinol (THC), are receiving growing attention in the scientific literature and in the media for their still undefined effects on human health. A substantial proportion of the population uses these products for recreational or medical uses, yet the extent to which they are safe or efficacious is still unclear, although preclinical studies indicate that some cannabinoids may have significant therapeutic value in many neurological diseases such as stroke. Indeed, a recent systemic review and meta-analysis [7] reported that cannabinoid agonists on CB1 and CB2 receptors significantly reduce the infarct volume in transient and permanent ischemia and improve early and late functional outcomes in experimental stroke when given after stroke onset. 

In this research, we studied the effects of CBD or THC administration on neuronal degeneration and glia activation in rat organotypic hippocampal slices exposed to oxygen glucose deprivation (OGD), an in vitro model of brain ischemia [30,31]. Our focus was to define the differential protective or damaging effects of CBD vs. THC and the possible involvement of TRPV2 channels in the mechanism of action of CBD and THC. Our results show that the two cannabinoids produced differential effects on OGD-induced damage to CA1 pyramidal neurons: CBD was neuroprotective while THC exacerbated the neuronal degeneration induced by OGD. The neuroprotective effect of CBD depended, at least in part, on TRPV2 channel activation since it was blocked by the TRPV2 antagonist tranilast. Indeed, while in CBD-treated slices, the number of karyorrectic/karyolythic neuros was similar to controls, when the slices were incubated with CBD and tranilast, the number of damaged neurons was not different from that in OGD slices. Interestingly, we found that after OGD, the expression of the TRPV2 channel decreased significantly in CA1 pyramidal neurons, and CBD prevented this effect while THC did not. Nevertheless, qualitative analyses showed that OGD increased the expression of the TRPV2 channel in microglia cells, which changed their morphology to become activated-phagocytic and rod microglia [27]. CBD prevented all these effects while THC did not. 

Recent reports [32,33,34] quantified the potency of THC and CBD contents in street cannabis and found that the median content of THC is significantly higher than 10 years ago, while the content of CBD is very low. The growing popularity of recreational consumption of cannabis, especially among the young population, raises concerns about its safety since cannabis variants containing higher THC contents pose significant physical and mental health risks, particularly for those susceptible to its harmful effects, and can lead to substance dependency [35,36]. Furthermore, ischemic stroke is the most commonly reported adverse neurovascular effect of cannabis use in the young population [35]. One of the mechanisms that may cause stroke in young cannabis users is the generation of reactive oxygen species that cause oxidative stress, one of the known mechanisms of stroke [35]. Additionally, we had previously demonstrated that in slices treated with CBD in combination with THC, when simulating different types of mixtures of the two compounds that can be found in different products or in plants, THC counteracted the neuroprotective effect of CBD [23], posing further health concerns surrounding the use of preparations in which the two compounds are present at different, often unknown, concentrations. 

In in vitro and in vivo models of ischemia, we previously identified and quantified different forms of damaged neurons, such as pyknotic neurons, defined as high-density nucleus (HDN) neurons, and karyorrectic/karyolythic neurons, defined low-density nucleus (LDN) neurons [23,37,38,39]. Pyknosis usually precedes karyorrhexis and karyolysis and can occur as a result of programmed cell death, cell senescence, or necrosis [40]. In CA1 of OGD slices, we had previously demonstrated [23] that OGD significantly increases both HDN and LDN damaged neurons. In this study we confirmed that CBD prevented the neurodegeneration caused by OGD, thereby significantly decreasing LDN neurons, and the interesting finding obtained in this work is that the neuroprotective effect of CBD was blocked by the TRPV2 antagonist tranilast. 

Previous preclinical studies demonstrated that the non-psychotomimetic cannabinoid CBD induces protective effects on neural degeneration [23,41]. Interestingly, repeated treatment with CBD improves functional outcome and survival rates after an infarct, suggesting that it may have neuroprotective effects not only at the early phase but also at late time points after the ischemic insult [42]. Multiple receptors, which cause a combination of antioxidant and anti-inflammatory effects [43], have been proposed to mediate the neuroprotective effects of CBD. *Cannabis sativa* compounds have proven to be among the most potent, although non-specific, TRPV2 channel activators, with an EC50 in the micromolar range [12,14]. However, CBD is also a potent activator of TRPV1 and TRPA1 channels [12]. In this study, we observed for the first time that the neuroprotective effects of CBD were dependent, at least in part, on TRPV2 receptor activation since the TRPV2 antagonist tranilast [44,45,46] attenuated the neuroprotection brought about by CBD. The expression of the TRPV2 channel, present in control conditions mainly in CA1 pyramidal neurons, decreased significantly after OGD, possibly mirroring the damage to neurons. Nevertheless, after the incubation of the slices with CBD, the expression of TRPV2 was similar to that in control slices, but in OGD slices treated with CBD, the channel was mainly expressed in non-neuronal cells. For the first time, a detailed colocalization analysis demonstrated that, in our experimental conditions, TRPV2 channel expression increased in microglia cells, while astrocytes did not express TRPV2 in any of our experimental groups. After OGD, the expression of the TRPV2 channel in CA1 SP was increased mainly in activated, phagocytic microglia cells, the density of which increased significantly in comparison to controls. Our findings are in accordance with those obtained in models of cardiac infarction in which increases in TRPV2 mRNA [47] or protein [48] were found in macrophages. It has previously been shown that CBD enhances TRPV2 protein expression and promotes its translocation to the cell surface of BV-2 cells in culture [24], predominantly in the membrane fraction. In addition, CBD affects some microglial functions and also modulates many microglial genes involved in the regulation of stress responses and inflammation [22], and consequently central anti-inflammatory properties. CBD successfully attenuates neuroinflammation while simultaneously improving mitochondrial function and ATP production via TRPV2 activation in mouse primary neurons, microglia cultures, and AD mice models [49]. The physiological role of TRPV2 is probably one of the most controversial among TRP channels [50]. Indeed, it has been shown that TRPV2 activation promotes increased expression of the channel itself. Somehow this finding is counterintuitive since, on TRPV2 channel activation, downregulation of the channel that limits Ca^2+^ entry may be the expected outcome. It has been shown that TRPV2 plays a key role in macrophage and microglia phagocytosis [24,51] and, since TRPV2 channels are mechanoreceptors, it is possible that phagocytosis itself, by stressing the cell membrane, activates the channel, thus engaging the amplification process. Thus, TRPV2 channels seem to be part of a feed-forward amplification of intracellular Ca^2+^ signaling that enhances microglia activation [24]. Nevertheless, we found that phagocytic microglia increase significantly after OGD, while CBD, but not THC, significantly decreased the number of phagocytic cells, which remained significantly higher than in control slices. However, the possibility that the protective effect of CBD is also mediated by receptors other than the TRPV2 channel should be taken into consideration.

Microglial morphological characteristics vary with time and space, and with environmental factors that affect the phagocytic function in different brain states, from physiology to pathology. So far, there is no definitive concordance on whether microglia phagocytosis of neurons or neuronal debris is beneficial or detrimental to brain repair. Efficient clearance of tissue debris is considered fundamental to the reconstruction and reorganization of brain tissue and neuronal networking after an injury [52,53,54], and participation of microglia in debris clearance takes place at the early stage after the insult [55].

It has been shown that microglia can undergo morphofunctional modifications that cause the cell to elongate to form rod microglia [27,29,56,57,58,59,60]. In this study, we found that in response to ischemia, microglia rapidly modified their morphology to rod–like cells linked head-to-tail to form trains over 100 µm long, which spanned from the SR toward CA1 [27]. Quantitative analysis showed that the increase in rod microglia formation in CA1 SP was opposite but symmetrical to the formation of phagocytic microglia. Indeed, rod microglia increased more in CBD-treated slices after OGD than in OGD slices themselves. Trains of rod microglia are supposed to migrate to the areas affected by the damage, where they acquire the amoeboid phagocytic form and eliminate the damaged neurons by phagocytosis [27,61,62,63]. If the tissue is not completely damaged, phagocytic microglia, after fulfilling their functions, are able to return to their resting state and continue patrolling the tissue [63]. The 30 min duration of OGD is considered a very strong ischemic insult and our results confirm the findings by Graeber and Mehraein (1994) [64], who described that when the tissue is highly damaged and with extensive neuronal necrosis, microglia acquire the morphology of activated, phagocytic cells. Indeed, in slices that were completely damaged after OGD, we found a highly significant increase in phagocytic microglia, while when CBD exerted its protective effect from the insult, rod microglia were highly present. It is now more and more evident that many microglia activation states exist, and it is possible that too much microglia activation is deleterious for neurons [65,66,67,68,69,70,71].

In this and other works [23,39], we have also demonstrated that THC has negative effects on neuronal survival after ischemia, and THC does not show any of the effects seen after CBD administration on the TRPV2 channel, microglia phagocytosis, or rod cell formation. It can be postulated that, when using different preparations of cannabinoids with diverse contents of CBD and THC, the positive effects of CBD might be counteracted by the high levels of THC present. 

In conclusion, our results show that cannabinoids, challenged in an in vitro model of cerebral ischemia, exert neurotoxic or neuroprotective effects, which depend on the cannabinoid used, with CBD showing protective while THC showing damaging properties. The effect of CBD was related to its agonistic activity on the TRPV2 channel. The neuroprotective effects of CBD and products containing high proportions of it, combined with its excellent tolerability, make CBD a promising candidate for clinical studies and future therapeutic use. Nevertheless, it must be kept in mind that the positive effects of CBD may be counteracted by the negative effects caused by high levels of THC. These results caution against the indiscriminate use of cannabis or cannabinoid preparations for recreational or therapeutic use.

## 4. Materials and Methods

### 4.1. Animals

Male and female Wistar rat pups (7–9 days old, Charles River, MI, Italy) were housed at 23 ± 1 °C under a 12 h light–dark cycle (lights on at 07:00) and were fed a standard laboratory diet with ad libitum access to water. Animal use procedures were in accordance with the National Institutes of Health Guide for the Care and Use of Laboratory Animals (NIH publications no. 80-23, revised 1996). The experimental protocols were approved by the Animal Care Committee of the Department of Health Sciences, University of Florence (17E9C.N.GSO/2021), in compliance with the European Convention for the Protection of Vertebrate Animals used for Experimental and Other Scientific Purposes (ETS No. 123) and the European Communities Council Directive of 24 November 1986 (86/609/EEC). The authors further attest that all efforts were made to minimize the number of animals used and their suffering. 

### 4.2. Materials

Cannabidiol (CBD) was purchased from Tocris Cookson (Bristol, UK). Tissue culture reagents were purchased from Gibco-BRL (San Giuliano Milanese, MI, Italy) and Sigma (St. Louis, MO, USA). Δ9-tetrahydrocannabinol (THC) and tranilast (TNL) were purchased from Sigma. The authorization number for the scientific use of THC was n. SP/94, of 07.07.2021.

### 4.3. Oxygen and Glucose Deprivation (OGD) in Rat Organotypic Hippocampal Slices and Drug Incubation

Briefly, organotypic hippocampal slice cultures were prepared as described [72]. Hippocampi were removed from the brains of male and female 7–9-day-old Wistar rats. Transverse slices (420 μm thick) were cut with a McIlwain tissue chopper, quickly transferred to semiporous membranes inserts, and maintained in culture for 14 days in vitro. Oxygen glucose deprivation (OGD) was delivered by incubating the slices with pre-saturated (95% N2/5% CO_2_) glucose-free and serum-free medium for 30 min at 37 °C in an airtight anoxic chamber. The slices were then transferred to oxygenated serum-free medium containing 5 mg/mL glucose, and returned to the incubator under normoxic conditions to recover for 24 h [30,31]. The drugs were incubated during the 30 min of OGD and in the subsequent 24 h of recovery. THC was dissolved in methanol and stored at −20 °C. CBD was dissolved in dimethyl sulfoxide (DMSO) and stored at −20 °C. Slices were incubated with the TRPV2 antagonist TNL at a concentration of 50 µM. Drugs were diluted in cell culture medium to the final concentration just before their application to hippocampal slices, with a maximal final solvent concentration of 0.1% (*v*/*v*) DMSO or 0.3% (*v*/*v*) methanol. Control experiments were performed with final concentrations of DMSO and methanol or methanol alone as in treated slices. No significant effects of the solvents on the parameters investigated were ever found. Four experimental groups were carried out: (1) control slices (CTR); (2) slices exposed for 30 min with oxygen-glucose deprivation for 30 min (OGD); (3) slices exposed for 30 min to oxygen-glucose deprivation and incubated during the 30 min of OGD and in the subsequent 24 h of recovery with 10 µM CBD (OGD + CBD); (4) slices exposed for 30 min to oxygen-glucose deprivation and incubated during the 30 min of OGD and in the subsequent 24 h of recovery with 1 µM THC (OGD + THC).

### 4.4. Neuronal Cell Death Evaluation by Propidiom Iodide

Cell death was evaluated by incubating the slices with propidium iodide (PI, 5 µg/mL). Thirty minutes later, fluorescence was visualized using an inverted fluorescence microscope (Olympus IX-50; Solent Scientific, Segensworth, UK) equipped with a xenon-arc lamp, and a rhodamine filter. Images were digitized using a video image obtained by a CCD camera (Diagnostic Instruments Inc., Sterling Heights, MI, USA) controlled by software (InCyt Im1TM; Intracellular Imaging Inc., Cincinnati, OH, USA), and subsequently analyzed using the Image-Pro Plus morphometric analysis software (Media Cybernetics, Silver Spring, MD, USA).

### 4.5. Immunohistochemistry

Immunohistochemistry was performed with the free-floating method [73,74] as previously described. The protocols used for the different immunostaining approaches and the antibodies used are reported below. Washing was always for 3 × 5 min in PBS-TX (0.3% Triton X-100 in PBS).

#### 4.5.1. NeuN + TRPV2 + GFAP Triple-Labeling Immunostaining

Day 1. Organotypic hippocampal slices were placed in 24-well plates, washed in PBS-TX, and blocked for 60 min with blocking buffer (BB, 10% normal goat serum in PBS-TX). Slices were then incubated overnight at 4 °C under slight agitation in a solution with two primary antibodies, a rabbit anti-TRPV2 antibody (1:200 in BB, product code: ab6183, Abcam, Cambridge, UK) and a mouse anti-NeuN antibody to immunostain the neurons (1:400 in BB, product code: MAB377, Millipore, Billerica, MA, USA).

Day 2. After extensive washing, the sections were incubated for 2 h at room temperature in the dark with AlexaFluor 635 goat anti-rabbit secondary antibody (1:400 in BB, product code: A31577, Thermo Fisher Scientific, Waltham, MA, USA), and then for 2 h at room temperature in the dark with AlexaFluor 555 donkey anti-mouse (1:400 in BB, product code: A31570, Thermo Fisher Scientific) and AlexaFluor 635 goat anti-rabbit (1:400 in BB). After washing, the astrocytes were immunostained using a mouse anti-GFAP antibody conjugated with the fluorochrome AlexaFluor 488 (1:500 in BB, product code: MAB3402X, Millipore, Billerica, MA, USA).

#### 4.5.2. NeuN + TRPV2 + IBA1 Triple-Labeling Immunostaining

Day 1. Organotypic hippocampal slices were washed in PBS-TX, blocked with BB (see above), and then incubated overnight at 4 °C under slight agitation in a solution with three primary antibodies: a rabbit anti-TRPV2 antibody (1:200 in BB), a mouse anti-NeuN antibody to immunostain the neurons (1:400 in BB), and a goat anti-IBA1 antibody (1:200, product code: 011-27991, WAKO, Osaka, Japan). 

Day 2. After extensive washing, the slices were incubated for 2 h at room temperature in the dark with AlexaFluor 488 donkey anti-rabbit secondary antibody (1:400 in BB, product code: A21206 Thermo Fisher), then after washing, for 2 h at room temperature in the dark with AlexaFluor 635 donkey anti-goat antibody (1:400 in BB, product code: A21082 Thermo Fisher) and AlexaFluor 488 donkey anti-rabbit antibody (1:400 in BB), and finally, after washing, for 2 h at room temperature in the dark with AlexaFluor 555 donkey anti-mouse antibody (1:400 in BB), AlexaFluor 635 donkey anti-goat antibody (1:400 in BB), and AlexaFluor 488 donkey anti-rabbit antibody (1:400 in BB).

After washing, the sections were mounted on gelatin-coated slides using Vectashield mounting medium with DAPI (H-1200, Vectashield, Burlingame, CA, USA).

### 4.6. Microscopy Techniques and Qualitative and Quantitative Analyses

Confocal microscopy acquisitions were performed in the regions of interest (ROIs), CA1 Str. Pyramidalis (CA1 SP) and CA1 Str. Radiatum (CA1 SR), of the dorsal hippocampus. 

Slices were observed under a LEICA TCS SP7 confocal laser scanning microscope (Leica Microsystems CMS GmbH) equipped with a 20× objective and a 63× objective. The parameters of acquisition were maintained at a constant. With the 20× objective: frame dimension 1024 × 1024 pixels, frequency of acquisition 200 Hz, z-step 1.2 μm. With the 63× objective: frame dimension 1024 × 1024 pixels, frequency of acquisition 200 Hz, z-step 0.3 μm. 

Quantitative analyses of neurons were performed on stacks of 10 consecutive confocal z-scans of the NeuN immunofluorescence channel (1.2 µm each, total thickness 12 µm, acquired with the 20× objective) using ImageJ software (National Institute of Health, http://rsb.info.nih.gov/ij, accessed on 1 July 2021).

Areas of region of interest (CA1 SP and CA1 SR) were calculated in mm^2^, and the density of immunopositive LDN neurons (low-density nucleus neurons) was calculated in cells/mm^2^. LDN neurons are characterized by karyorrhexis, which represents an index of damaged nuclei. In LDN neurons, NeuN immunostaining is faint or absent in the nucleus, while it persists in the cytoplasm [37]. 

Quantitative analyses of TRPV2 expression was performed using the threshold function of ImageJ. Quantitation of immunofluorescence was then obtained from the ratio between positive pixels above the threshold and total pixels in each region of interest, which was expressed as a percentage.

Quantitative analyses of astrocytes’ density (cells/mm^2^), GFAP expression (percentage of GFAP+ pixels in ROI), and GFAP expression per cell (GFAP+ pixels in ROI/cell) were performed on stacks of 10 consecutive confocal z-scans of the GFAP immunofluorescence channel (1.2 µm each, total thickness 12 µm, acquired with the 20× objective). GFAP+ pixels were selected by setting the threshold level to the GFAP signal with ImageJ software.

Quantitative analyses of the density of total microglia, rod microglia, and phagocytic microglia (cells/mm^2^) were performed on stacks of three consecutive confocal z-scans of the IBA1 immunofluorescence channel (1.2 µm each, total thickness 3.6 µm, acquired with the 20× objective).

Rod microglia are characterized by an elongated, rod-like morphology and a length that varies between 40 µm and above 100 µm. Rod microglia often form “trains” in which two or more rod microglia cells are linked through “head-to-tail” contact (see also [27]). A microglia cell was considered phagocytic if IBA1 was colocalized with NeuN immunostaining.

### 4.7. Statistical Analysis

Data are presented as the means ± SEM of the experiments. Immunohistochemistry data were statistically analyzed by one-way analysis of variance (ANOVA) followed by a Newman–Keuls multiple comparisons test. All statistical calculations were performed using GraphPad Prism v.8 for Windows (GraphPad Software, San Diego, CA, USA). A probability value of *p* < 0.05 was considered significant.

## 5. Conclusions

In conclusion, our results show that cannabinoids, challenged in an in vitro model of cerebral ischemia, exert neurotoxic or neuroprotective effects, which depend on the cannabinoid used, with CBD showing protective and THC showing damaging properties. The effect of CBD was related to its agonistic activity on the TRPV2 channel. The neuroprotective effects of CBD and products containing high proportions of it, combined with its excellent tolerability, make CBD a promising candidate for clinical studies and future therapeutic use. Nevertheless, it must be remembered that the positive effects of CBD may be counteracted by the negative effects caused by high levels of THC. These results caution against the indiscriminate use of cannabis or cannabinoid preparations for recreational or therapeutic use.

## Data Availability

Not applicable.

## References

[1] Tomari S., Tanaka T., Ihara M., Matsuki T., Fukuma K., Matsubara S., Nagatsuka K., Toyoda K. (2017). Risk factors for post-stroke seizure recurrence after the first episode. Seizure.

[2] DECRETO 9 Novembre 2015. Funzioni Di Organismo Statale Per La Cannabis Previsto Dagli Articoli 23 E 28 Della Convenzione Unica Sugli Stupefacenti Del 1961, Come Modificata Nel 1972. (15A08888). https://www.gazzettaufficiale.it/eli/id/2015/11/30/15A08888/sg.

[3] Turner S.E., Williams C.M., Iversen L., Whalley B.J. (2017). Molecular Pharmacology of Phytocannabinoids. Prog. Chem. Org. Nat. Prod..

[4] Mechoulam R., Hanuš L.O., Pertwee R., Howlett A.C. (2014). Early phytocannabinoid chemistry to endocannabinoids and beyond. Nat. Rev. Neurosci..

[5] Hind W.H., England T.J., O’Sullivan S.E. (2016). Cannabidiol protects an in vitro model of the blood-brain barrier from oxygen-glucose deprivation via PPARγ and 5-HT1A receptors. Br. J. Pharmacol..

[6] Alvarez F.J., Lafuente H., Rey-Santano M.C., Mielgo V.E., Gastiasoro E., Rueda M., Pertwee R.G., Castillo A.I., Romero J., Martínez-Orgado J. (2008). Neuroprotective effects of the nonpsychoactive cannabinoid cannabidiol in hypoxic-ischemic newborn piglets. Pediatr. Res..

[7] England T.J., Hind W.H., Rasid N.A., O’Sullivan S.E. (2015). Cannabinoids in experimental stroke: A systematic review and meta-analysis. J. Cereb. Blood Flow Metab..

[8] Rumalla K., Reddy A.Y., Mittal M.K. (2016). Recreational marijuana use and acute ischemic stroke: A population-based analysis of hospitalized patients in the United States. J. Neurol. Sci..

[9] Chesney E., Oliver D., Green A., Sovi S., Wilson J., Englund A., Freeman T.P., McGuire P. (2020). Adverse effects of cannabidiol: A systematic review and meta-analysis of randomized clinical trials. Neuropsychopharmacology.

[10] Howlett A.C. (2002). The cannabinoid receptors. Prostaglandins Other Lipid Mediat..

[11] Pertwee R.G. (2008). The diverse CB1 and CB2 receptor pharmacology of three plant cannabinoids: Delta9-tetrahydrocannabinol, cannabidiol and delta9-tetrahydrocannabivarin. Br. J. Pharmacol..

[12] De Petrocellis L., Ligresti A., Moriello A.S., Allarà M., Bisogno T., Petrosino S., Stott C.G., Di Marzo V. (2011). Effects of cannabinoids and cannabinoid-enriched Cannabis extracts on TRP channels and endocannabinoid metabolic enzymes. Br. J. Pharmacol..

[13] Soethoudt M., Grether U., Fingerle J., Grim T.W., Fezza F., De Petrocellis L., Ullmer C., Rothenhäusler B., Perret C., Van Gils N. (2017). Cannabinoid CB 2 receptor ligand profiling reveals biased signalling and off-target activity. Nat. Commun..

[14] Qin N., Neeper M.P., Liu Y., Hutchinson T.L., Lubin M.L., Flores C.M. (2008). TRPV2 is activated by cannabidiol and mediates CGRP release in cultured rat dorsal root ganglion neurons. J. Neurosci..

[15] Landucci E., Pellegrini-Giampietro D.E., Gianoncelli A., Ribaudo G. (2022). Cannabidiol preferentially binds TRPV2: A novel mechanism of action. Neural Regen. Res..

[16] De Lago E., De Miguel R., Lastres-Becker I., Ramos J.A., Fernández-Ruiz J. (2004). Involvement of vanilloid-like receptors in the effects of anandamide on motor behavior and nigrostriatal dopaminergic activity: In vivo and in vitro evidence. Brain Res..

[17] Nedungadi T.P., Dutta M., Bathina C.S., Caterina M.J., Cunningham J.T. (2012). Expression and distribution of TRPV2 in rat brain. Exp. Neurol..

[18] Wainwright A., Rutter A.R., Seabrook G.R., Reilly K., Oliver K.R. (2004). Discrete expression of TRPV2 within the hypothalamo-neurohypophysial system: Implications for regulatory activity within the hypothalamic-pituitary-adrenal axis. J. Comp. Neurol..

[19] Luo H., Rossi E., Saubamea B., Chasseigneaux S., Cochois V., Choublier N., Smirnova M., Glacial F., Perrière N., Bourdoulous S. (2019). Cannabidiol Increases Proliferation, Migration, Tubulogenesis, and Integrity of Human Brain Endothelial Cells through TRPV2 Activation. Mol. Pharm..

[20] Luo H., Saubamea B., Chasseigneaux S., Cochois V., Smirnova M., Glacial F., Perrière N., Chaves C., Cisternino S., Declèves X. (2020). Molecular and Functional Study of Transient Receptor Potential Vanilloid 1-4 at the Rat and Human Blood-Brain Barrier Reveals Interspecies Differences. Front. Cell Dev. Biol..

[21] Santoni G., Amantini C. (2019). The Transient Receptor Potential Vanilloid Type-2(TRPV2) Ion Channels in Neurogenesis andGliomagenesis: Cross-Talk between Transcription Factors and Signaling Molecules. Cancers.

[22] Juknat A., Rimmerman N., Levy R., Vogel Z., Kozela E. (2012). Cannabidiol affects the expression of genes involved in zinc homeostasis in BV-2 microglial cells. Neurochem. Int..

[23] Landucci E., Mazzantini C., Lana D., Davolio P.L., Giovannini M.G., Pellegrini-Giampietro D.E. (2021). Neuroprotective Effects of Cannabidiol but Not Δ 9-Tetrahydrocannabinol in Rat Hippocampal Slices Exposed to Oxygen-Glucose Deprivation: Studies with Cannabis Extracts and Selected Cannabinoids. Int. J. Mol. Sci..

[24] Hassan S., Eldeeb K., Millns P.J., Bennett A.J., Alexander S.P.H., Kendall D.A. (2014). Cannabidiol enhances microglial phagocytosis via transient receptor potential (TRP) channel activation. Br. J. Pharmacol..

[25] Hulse R.E., Winterfield J., Kunkler P., Kraig R.P. (2001). Astrocytic Clasmatodendrosis in Hippocampal Organ Culture. Glia.

[26] Mercatelli R., Lana D., Bucciantini M., Giovannini M.G., Cerbai F., Quercioli F., Zecchi-Orlandini S., Delfino G., Wenk G.L., Nosi D. (2016). Clasmatodendrosis and b-amyloidosis in aging hippocampus. FASEB J..

[27] Lana D., Gerace E., Magni G., Cialdai F., Monici M., Mannaioni G., Giovannini M.G. (2022). Hypoxia/Ischemia-Induced Rod Microglia Phenotype in CA1 Hippocampal Slices. Int. J. Mol. Sci..

[28] Tam W.Y., Au N.P.B., Ma C.H.E. (2016). The association between laminin and microglial morphology in vitro. Sci. Rep..

[29] Ziebell J.M., Taylor S.E., Cao T., Harrison J.L., Lifshitz J. (2012). Rod microglia: Elongation, alignment, and coupling to form trains across the somatosensory cortex after experimental diffuse brain injury. J. Neuroinflamm..

[30] Gerace E., Landucci E., Scartabelli T., Moroni F., Chiarugi A., Pellegrini-Giampietro D.E. (2015). Interplay between histone acetylation/deacetylation and poly(ADP-ribosyl)ation in the development of ischemic tolerance in vitro. Neuropharmacology.

[31] Landucci E., Filippi L., Gerace E., Catarzi S., Guerrini R., Pellegrini-Giampietro D.E. (2018). Neuroprotective effects of topiramate and memantine in combination with hypothermia in hypoxic-ischemic brain injury in vitro and in vivo. Neurosci. Lett..

[32] Bonnet U., Preuss U. (2017). The cannabis withdrawal syndrome: Current insights. Subst. Abuse Rehabil..

[33] Volkow N.D., Baler R.D., Compton W.M., Weiss S.R.B. (2014). Adverse health effects of marijuana use. N. Engl. J. Med..

[34] Freeman T.P., Winstock A.R. (2015). Examining the profile of high-potency cannabis and its association with severity of cannabis dependence. Psychol. Med..

[35] Wolff V., Jouanjus E. (2017). Strokes are possible complications of cannabinoids use. Epilepsy Behav..

[36] Jouanjus E., Raymond V., Lapeyre-Mestre M., Wolff V. (2017). What is the Current Knowledge About the Cardiovascular Risk for Users of Cannabis-Based Products? A Systematic Review. Curr. Atheroscler. Rep..

[37] Fusco I., Ugolini F., Lana D., Coppi E., Dettori I., Gaviano L., Nosi D., Cherchi F., Pedata F., Giovannini M.G. (2018). The selective antagonism of adenosine A2B receptors reduces the synaptic failure and neuronal death induced by oxygen and glucose deprivation in rat CA1 hippocampus in vitro. Front. Pharmacol..

[38] Dettori I., Gaviano L., Ugolini F., Lana D., Bulli I., Magni G., Rossi F., Giovannini M.G., Pedata F. (2021). Protective effect of adenosine A2B receptor agonist, BAY60-6583, against transient focal brain ischemia in rat. Front. Pharmacol. Pharm..

[39] Landucci E., Mazzantini C., Lana D., Giovannini M.G., Pellegrini-Giampietro D.E. (2022). Neuronal and Astrocytic Morphological Alterations Driven by Prolonged Exposure with Δ9-Tetrahydrocannabinol but Not Cannabidiol. Toxics.

[40] Elmore S. (2007). Apoptosis: A review of programmed cell death. Toxicol. Pathol..

[41] Echeverry C., Prunell G., Narbondo C., de Medina V.S., Nadal X., Reyes-Parada M., Scorza C. (2021). A Comparative In Vitro Study of the Neuroprotective Effect Induced by Cannabidiol, Cannabigerol, and Their Respective Acid Forms: Relevance of the 5-HT 1A Receptors. Neurotox. Res..

[42] Hayakawa K., Irie K., Sano K., Watanabe T., Higuchi S., Enoki M., Nakano T., Harada K., Ishikane S., Ikeda T. (2009). Therapeutic time window of cannabidiol treatment on delayed ischemic damage via high-mobility group box1-inhibiting mechanism. Biol. Pharm. Bull..

[43] Hayakawa K., Mishima K., Fujiwara M. (2010). Therapeutic Potential of Non-Psychotropic Cannabidiol in Ischemic Stroke. Pharmaceuticals.

[44] Aoyagi K., Ohara-Imaizumi M., Nishiwaki C., Nakamichi Y., Nagamatsu S. (2010). Insulin/phosphoinositide 3-kinase pathway accelerates the glucose-induced first-phase insulin secretion through TrpV2 recruitment in pancreatic β-cells. Biochem. J..

[45] Hisanaga E., Nagasawa M., Ueki K., Kulkarni R.N., Mori M., Kojima I. (2009). Regulation of calcium-permeable TRPV2 channel by insulin in pancreatic beta-cells. Diabetes.

[46] Mihara H., Boudaka A., Shibasaki K., Yamanaka A., Sugiyama T., Tominaga M. (2010). Involvement of TRPV2 activation in intestinal movement through nitric oxide production in mice. J. Neurosci..

[47] Entin-Meer M., Levy R., Goryainov P., Landa N., Barshack I., Avivi C., Semo J., Keren G. (2014). The transient receptor potential vanilloid 2 cation channel is abundant in macrophages accumulating at the peri-infarct zone and may enhance their migration capacity towards injured cardiomyocytes following myocardial infarction. PLoS ONE.

[48] Entin-Meer M., Keren G. (2020). Potential roles in cardiac physiology and pathology of the cation channel TRPV2 expressed in cardiac cells and cardiac macrophages: A minireview. Am. J. Physiol. Heart Circ. Physiol..

[49] Yang S., Du Y., Zhao X., Tang Q., Su W., Hu Y., Yu P. (2022). Cannabidiol Enhances Microglial Beta-Amyloid Peptide Phagocytosis and Clearance via Vanilloid Family Type 2 Channel Activation. Int. J. Mol. Sci..

[50] Perálvarez-Marín A., Doñate-Macian P., Gaudet R. (2013). What do we know about the transient receptor potential vanilloid 2 (TRPV2) ion channel?. FEBS J..

[51] Link T.M., Park U., Vonakis B.M., Raben D.M., Soloski M.J., Caterina M.J. (2010). TRPV2 has a pivotal role in macrophage particle binding and phagocytosis. Nat. Immunol..

[52] Sierra A., Encinas J.M., Deudero J.J.P., Chancey J.H., Enikolopov G., Overstreet-Wadiche L.S., Tsirka S.E., Maletic-Savatic M. (2010). Microglia shape adult hippocampal neurogenesis through apoptosis-coupled phagocytosis. Cell Stem Cell.

[53] Weldon D.T., Rogers S.D., Ghilardi J.R., Finke M.P., Cleary J.P., O’Hare E., Esler W.P., Maggio J.E., Mantyh P.W. (1998). Fibrillar beta-amyloid induces microglial phagocytosis, expression of inducible nitric oxide synthase, and loss of a select population of neurons in the rat CNS in vivo. J. Neurosci..

[54] Ito U., Nagasao J., Kawakami E., Oyanagi K. (2007). Fate of disseminated dead neurons in the cortical ischemic penumbra: Ultrastructure indicating a novel scavenger mechanism of microglia and astrocytes. Stroke.

[55] Schilling M., Besselmann M., Müller M., Strecker J.K., Ringelstein E.B., Kiefer R. (2005). Predominant phagocytic activity of resident microglia over hematogenous macrophages following transient focal cerebral ischemia: An investigation using green fluorescent protein transgenic bone marrow chimeric mice. Exp. Neurol..

[56] Taylor S.E., Morganti-Kossmann C., Lifshitz J., Ziebell J.M. (2014). Rod microglia: A morphological definition. PLoS ONE.

[57] Yuan T.F., Liang Y.X., Peng B., Lin B., So K.F. (2015). Local proliferation is the main source of rod microglia after optic nerve transection. Sci. Rep..

[58] Rao Y., Liang Y.X., Peng B. (2017). A revisit of rod microglia in preclinical studies. Neural Regen. Res..

[59] Holloway O.G., Canty A.J., King A.E., Ziebell J.M. (2019). Rod microglia and their role in neurological diseases. Semin. Cell Dev. Biol..

[60] Giordano K.R., Denman C.R., Dubisch P.S., Akhter M., Lifshitz J. (2021). An update on the rod microglia variant in experimental and clinical brain injury and disease. Brain Commun..

[61] Nimmerjahn A., Kirchhoff F., Helmchen F. (2005). Neuroscience: Resting microglial cells are highly dynamic surveillants of brain parenchyma in vivo. Science.

[62] Davalos D., Grutzendler J., Yang G., Kim J.V., Zuo Y., Jung S., Littman D.R., Dustin M.L., Gan W.B. (2005). ATP mediates rapid microglial response to local brain injury in vivo. Nat. Neurosci..

[63] Morsch M., Radford R., Lee A., Don E.K., Badrock A.P., Hall T.E., Cole N.J., Chung R. (2015). In vivo characterization of microglial engulfment of dying neurons in the zebrafish spinal cord. Front. Cell. Neurosci..

[64] Graeber M.B., Mehraein P. (1994). Microglial rod cells. Neuropathol. Appl. Neurobiol..

[65] Liddelow S.A., Barres B.A. (2017). Reactive Astrocytes: Production, Function, and Therapeutic Potential. Immunity.

[66] Khakh B.S., Sofroniew M.V. (2015). Diversity of astrocyte functions and phenotypes in neural circuits. Nat. Neurosci..

[67] Ben Haim L., Rowitch D.H. (2016). Functional diversity of astrocytes in neural circuit regulation. Nat. Rev. Neurosci..

[68] Khakh B.S., Deneen B. (2019). The Emerging Nature of Astrocyte Diversity. Annu. Rev. Neurosci..

[69] Pestana F., Edwards-Faret G., Belgard T.G., Martirosyan A., Holt M.G. (2020). No longer underappreciated: The emerging concept of astrocyte heterogeneity in neuroscience. Brain Sci..

[70] Cerbai F., Lana D., Nosi D., Petkova-Kirova P., Zecchi S., Brothers H.M., Wenk G.L., Giovannini M.G. (2012). The Neuron-Astrocyte-Microglia Triad in Normal Brain Ageing and in a Model of Neuroinflammation in the Rat Hippocampus. PLoS ONE.

[71] Lana D., Iovino L., Nosi D., Wenk G.L., Giovannini M.G. (2016). The neuron-astrocyte-microglia triad involvement in neuroinflammaging mechanisms in the CA3 hippocampus of memory-impaired aged rats. Exp. Gerontol..

[72] Gerace E., Landucci E., Scartabelli T., Moroni F., Pellegrini-Giampietro D.E. (2012). Rat hippocampal slice culture models for the evaluation of neuroprotective agents. Methods Mol. Biol..

[73] Giovannini M.G., Scali C., Prosperi C., Bellucci A., Vannucchi M.G., Rosi S., Pepeu G., Casamenti F. (2002). β-amyloid-induced inflammation and cholinergic hypofunction in the rat brain in vivo: Involvement of the p38MAPK pathway. Neurobiol. Dis..

[74] Lana D., Melani A., Pugliese A.M., Cipriani S., Nosi D., Pedata F., Giovannini M.G. (2014). The neuron-astrocyte-microglia triad in a rat model of chronic cerebral hypoperfusion: Protective effect of dipyridamole. Front. Aging Neurosci..

